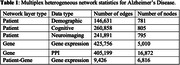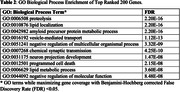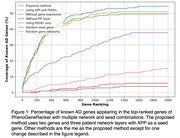# Alzheimer’s disease‐associated gene ranking using PhenoGeneRanker

**DOI:** 10.1002/alz.090044

**Published:** 2025-01-03

**Authors:** Most Tahmina Rahman, Fahad Saeed, Serdar Bozdag, Alzheimer’s Disease Neuroimaging Initiative

**Affiliations:** ^1^ University of North Texas, Denton, TX USA; ^2^ BioDiscovery Institute / University of North Texas, Denton, TX USA; ^3^ Florida International University, Miami, FL USA

## Abstract

**Background:**

Alzheimer’s disease (AD) is a neurogenerative disease that affect millions worldwide with no effective treatment. Several studies have been conducted to decipher to genomic underpinnings of AD. Due to its complex nature, many genes have been found to be associated with AD. Despite these findings, the pathophysiology of the disease is still elusive. In this study, we integrated multimodal gene and phenotype datasets of AD using network biology methods to prioritize potential AD‐related genes.

**Method:**

We collected phenotypic and genotypic datasets of AD from the Alzheimer’s Disease Neuroimaging Initiative (ADNI) database and protein‐protein interaction (PPI) dataset from STRING database. We constructed a multiplex heterogeneous network composed of patient and gene similarity networks. We built three phenotypic network layers utilizing the patient’s demographic, cognitive and neuroimaging data, and two gene similarity network layers using gene expression and PPI data (Table 1). Starting from AD‐associated gene APP, we applied PhenoGeneRanker to traverse this network to discover potential AD‐associated genes. To assess the impact of each network layer and seed gene, we also run PhenoGeneRanker on different variants of the network and seed genes. We performed Gene Ontology (GO) enrichment to assess the results.

**Result:**

We performed GO enrichment for the top 200 ranked genes. As a negative control, we repeated the same for the bottom 200 ranked genes. The top genes were enriched in terms such as amyloid precursor protein metabolic process, lipid metabolic process, and programmed cell death (Table 2), whereas bottom‐ranked genes had no GO enrichment. We also found some genes in the top 200 such as PSEN2, CLU, SORL1, PICALM, and APOE, which were identified as AD‐related genes in literature. Our top 2000 gene list captured over 50% of genes that were reported as AD‐associated (Figure 1). Ablation studies were performed to assess the impact of each network layer and seed genes. We observed that impact of PPI layer and APP seed gene were high.

**Conclusion:**

We identified candidate genes for AD that were enriched in disease‐related GO terms and captured previously reported genes, which suggest that other top‐ranked genes could be novel AD‐associated genes.